# Molecular Characterization of Methicillin Resistant *Staphylococcus aureus* in West Bank-Palestine

**DOI:** 10.3389/fpubh.2019.00130

**Published:** 2019-05-28

**Authors:** Etaf Hadyeh, Kifaya Azmi, Rania Abu Seir, Inas Abdellatief, Ziad Abdeen

**Affiliations:** ^1^Al-Quds Public Health Society, Jerusalem, Palestine; ^2^Department of Medical Lab Sciences, Faculty of Health Professions, Jerusalem, Palestine; ^3^Faculty of Medicine, Al-Quds Nutrition and Health Research Institute, Al-Quds University, Jerusalem, Palestine; ^4^Biochemistry and Molecular Biology Department, Faculty of Medicine, Al-Quds University, Jerusalem, Palestine; ^5^Laboratory Department of Al-Makassed Charitable Hospital, Jerusalem, Palestine

**Keywords:** MRSA, *spa*, SCC*mec*, PVL, TSST-1, *arc*A, USA-300clone

## Abstract

**Background:** Methicillin-resistant *Staphylococcus aureus* (MRSA) is a public health threat and a major cause of hospital-acquired and community-acquired infections. This study aimed to investigate the genetic diversity of MRSA isolates from 2015 to 2017 and to characterize the major MRSA clones and anti-biogram trends in Palestine.

**Methodology:** Isolates were obtained from 112 patients admitted to different hospitals of West Bank and East Jerusalem, originating from different clinical sources. Antibiotic susceptibility patterns, staphylococcal chromosomal cassette *mec* (SCC*mec*) typing, and *Staphylococcus aureus* protein A (*spa*) typing were determined. Also, a panel of toxin genes and virulence factors was studied, including: Panton-Valentine Leukocidin (PVL), ACME-*arc*A, Toxic Shock Syndrome Toxin-1 (TSST-1), and Exfoliative Toxin A (ETA).

**Results:** Of the 112 confirmed MRSA isolates, 100% were resistant to all β-lactam antibiotics. Resistance rates to other non- β-lactam classes were as the following: 18.8% were resistant to trimethoprim-sulfamethoxazole, 23.2% were resistant to gentamicin, 34.8% to clindamycin, 39.3% to ciprofloxacin, and 63.4% to erythromycin. All MRSA isolates were susceptible to vancomycin (100%). Of all isolates, 32 isolates (28.6%) were multidrug- resistant (MDR). The majority of the isolates were identified as SCC*mec* type IV (86.6%). The molecular typing identified 29 *spa* types representing 12 MLST-clonal complexes (CC). The most prevalent *spa* types were: *spa* type t386 (CC1)/(12.5%), *spa* type t044 (CC80)/(10.7%), *spa* type t008 (CC8)/(10.7%), and *spa* type t223 (CC22)/(9.8%). PVL toxin gene was detected in (29.5%) of all isolates, while ACME-*arcA* gene was present in 18.8% of all isolates and 23.2% had the TSST-1 gene. The two most common *spa* types among the TSST-1positive isolates were the *spa* type t223 (CC22)/(Gaza clone) and the *spa* type t021 (CC30)/(South West Pacific clone). All isolates with the *spa* type t991 were ETA positive (5.4%). USA-300 clone (*spa* type t008, positive for PVL toxin gene and ACME-*arcA* genes) was found in nine isolates (8.0%).

**Conclusions:** Our results provide insights into the epidemiology of MRSA strains in Palestine. We report a high diversity of MRSA strains among hospitals in Palestine, with frequent SCC*mec* type IV carriage. The four prominent clones detected were: t386-IV/ CC1, the European clone (t044/CC80), Gaza clone (t223/CC22), and the USA-300 clone (t008/CC8).

## Introduction

Methicillin-resistant *Staphylococcus aureus* (MRSA) is an important bacterial pathogen in both community and healthcare-related settings in different parts of the world. It is one of the major human pathogens that can cause broad variety of human diseases ranging from mild skin and soft tissue infections (SSTIs) to severe life-threatening invasive infections; such as: endocarditis, osteomyelitis, necrotizing pneumonia, bacteremia, septicemia, meningitis, food poisoning, and toxic shock syndrome (1–5). Methicillin resistance is caused by an alteration in the penicillin-binding protein (PBP2a) which has a lower affinity to the β-lactam antibiotics; including: all penicillins, all cephalosporins (except the fifth generation ceftaroline), and carbapenems (6, 7). It is encoded by the *mecA* gene carried on a staphylococcal chromosomal cassette (SCC*mec*).Thus; the therapeutic options of MRSA strains are lowered and limited.

Epidemiologic typing and molecular characterization of MRSA are crucial to monitor the occurrence and development of new epidemic clones and to determine intervention policies (8–10). Since hospital-associated (HA-MRSA) clones differ from community-associated (CA-MRSA) clones, clonal characterization is important to determine the source and the transmission routes of MRSA strains. Different typing methods are used to characterize different MRSA strains and clones. Specifically, defining the Staphylococcal Chromosomal Cassette (SCC*mec*) type is important to suggest the origin of the clone either hospital acquired or community acquired. The SCC*mec* types: I, II, and III are associated with HA-MRSA strains while types IV and V are considered as community associated MRSA strains (11, 12). *Staphylococcus aureus* protein A (*spa*) typing is also frequently used and based on typing of protein A. The *spa* typing technique depends on DNA sequencing of short sequence repeats (SSRs) of the polymorphic conserved X region of the staphylococcal protein A gene (13). Additionally, defining which virulence factors a strain may harbor is important not only for understanding the virulence of the strain, but also for studying the epidemiology of different common clones. Among these are: Panton-Valentine Leukocidin toxin (PVL); a pore-forming cytotoxin that can highly cause leukocyte destruction and tissue necrosis (14), Toxic Shock Syndrome Toxin-1 (TSST-1); a superantigen that can mediate fever, hypotension, rash, multi-organ dysfunction, and lethal shocks (15), Exfoliative Toxin A (ETA); a toxin that can lead to hydrolysis of the superficial skin layers leading to cutaneous infections and staphylococcal scalded skin syndrome (SSSS) (16) and *arc*A; a surrogate marker for Arginine Catabolic Mobile Element (ACME) I. The ACME is related with pathogenicity of the MRSA isolates which enhances both virulence and the ability of MRSA to colonize human skin (17). MRSA is a major public health concern in Palestine and little is known and studied about its molecular epidemiology and common epidemic clones (18, 19). This study aimed to investigate the genetic diversity and virulence genes of MRSA isolates identifying the prominent clones causing invasive infections in healthcare settings in the West Bank-Palestine, from 2015 to 2017.

## Methodology

### Bacterial Strains and Data Collection

In the present cross sectional descriptive study, a total of 112 MRSA isolates were collected from different sources, including: wound, blood, nasal swabs, urine, pus, tissue, abscesses, ear swabs, sputum, and other clinical sources; such as: cerebrospinal fluid (CSF) culture, central venous pressure (CVP) tip culture, skin swabs, synovial fluid culture, and trap. The study period was between 16th of November 2015 and 13th of July 2017 collected from different hospitals distributed in the West Bank-Palestine. Demographic and clinical data including: age, sex, place of residence, date of administration and hospitalization, type of infection, isolate antimicrobial susceptibility testing, specimen origin, and date of isolation were collected from medical records. Age was categorized to four age groups, as the following: infant; from 0 to 1 year, children; from 1 to 10 years, adolescent; from 10 to 19 years and as adult; more than 19 years.

### Bacterial Culture and Identification of MRSA Strains

In the Central Laboratory of Al-Quds Public Health Society, MRSA isolates were identified phenotypically by colony morphology on blood agar and coagulase mannitol salt agar base (CMSA), gram stain, catalase, and coagulase tests. The methicillin resistant strains were defined by the disk agar diffusion method using a cefoxitin disk/30 μg. In addition, the MRSA strains were confirmed by the detection of *mec*A gene using PCR as described by Geha et al. (20).

### Antimicrobial Susceptibility Testing

The antimicrobial susceptibility testing was performed using a disk diffusion method according to CLSI (2017) recommendations (21). Eight antimicrobial agents were tested, as the following: penicillin G (10 units/disc), cefoxitin (30 μg), vancomycin (30 μg), erythromycin (15 μg), clindamycin (10 μg), and gentamicin (10 μg), and trimethoprim-sulfamethoxazole (25 μg), and ciprofloxacin (5 μg) were determined. Isolates were defined as multidrug resistance strains (MDR) when they were resistant to at least three different antibiotic groups in addition to resistance to the β-lactam antibiotics (22). The *D*-test was performed to test the inducible resistance to clindamycin as needed.

### DNA Extraction and Quantification

Genomic DNA was extracted from overnight fresh pure cultures on blood agar using DNA extraction kit (Nucleospin, Macherey-Nagel, Düren, Germany) according to the manufacturer's instructions (23). Isolates were freezed upon collection, and then thawed and sub-cultured on BA overnight to be used, later on, for genomic extraction.

### Molecular Characterization of MRSA Strains

#### PCR Identification of Staphylococcal Cassette Chromosome (SCCmec) Types

SCC*mec* types (I–V) were identified by multiplex PCR as described by Boye et al. (12). The primers used in this multiplex PCR assay are shown in Table 1.

**Table 1:** All target genes and primers used in this study.

**#**	**Gene**	**Oligo^a^Name**	**Primer sequence 5^**′**^-3^**′**^**	**Length (bp)**	**Reference^b^**
					
1	*mec*A	*mec*A1F^c^	GTAGAAATGACTGAACGTCCGATAA	310	(20)
		*mec*A2R^d^	CCAATTCCACATTGTTTCGGTCTAA		
2	SCC*mec* multiplex ″4 primers″	β/ccrA2F-B	ATTGCCTTGATAATAGCCYTCT	937	(24)
		α3/ccrA2R-B	TAAAGGCATCAATGCACAAACACT		
		ccrCF/ccrC	CGTCTATTACAAGATGTTAAGGATAAT	518	(24)
		ccrCR/ccrC	CCTTTATAGACTGGATTATTCAAAATA		
		1272F1/IS1272	GCCACTCATAACATATGGAA	415	(12)
		1272R1/IS1272	CATCCGAGTGAAACCCAAA		
		5R*mec*AF/IS431	TATACCAAACCCGACAACTAC	359	
		5R431R/IS431	CGGCTACAGTGATAACATCC		
3	*Spa*	1095F/*spa*-F	AGACGATCCTTCGGTGAGC	200–400	(25)
		1017R/*spa*-R	GCTTTTGCAATGTCATTTACTG		
4	PVL	luk-PV-1F/PVL	ATCATTAGGTAAAATGTCTGGACATGATCCA	433	(26)
		luk-PV-2R/PVL	GCATCAASTGTATTGGATAGCAAAAGC		
5	TSST-1	G*TSST*R-1F	ACCCCTGTTCCCTTATCATC	350	(27)
		G*TSST*R-2R	TTTTCAGTATTTGTAACGCC		
6	ETA	GETAR-1F	GCAGGTGTTGATTTAGCATT	93	(27)
		GETAR-2R	AGATGTCCCTATTTTTGCTG		
7	ACME*-arc*A	*arc*A-F	GAGCCAGAAGTACGCGAG	724	(28)
		*arc*A-R	CACGTAACTTGCTAGAACGAG		

a *Oligo, Oligonecleotide*.

b *Reference*.

c *F, Forward primer*.

d *R, reverse primer*.

#### Identification of spa Types

All MRSA isolates were characterized by *spa* sequence-based typing using the PCR primers and cycling as previously described by Shopsin et al. (Tables 1, 2) (25). All *spa* amplicons were sequenced (Hylabs, Rehovot, Israel) and analyzed using the *spa* Type Finder/Identifier: (http://spatyper.fortinbras.us/). Then, the Based Upon Repeat Pattern (BURP) algorithm of the Staph-Type™ software (Ridom GmbH, Münster, Germany) was used for clustering of *spa* types and grouping in *spa*-clonal complexes (*spa*-CC).

**Table 2:** Thermal cycler programs used for the amplification of the targeted genes and toxins in this study.

**#**	**Target Gene**	**PCR program (Temp^a^°C/Time)-35 cycles**					**Reference**
		**Initial denaturation**	**Denaturation**	**Annealing**	**Extension**	**Final extension**	
1	*mec*A	95°C/5 min^b^	95°C/30 s^c^	58°C/30 s	72°C/80 s	72°C/10 min	(20)
2	SCC*mec*	95°C /5 min	94°C/30 s	55°C/30 s	72°C/80 s	72°C/10 min	(12)
3	*spa*	95°C/5 min	95°C/30 s	58°C/30 s	72°C/45 s	72°C/10 min	(25)
4	*lukS/lukF*	95°C/5 min	95°C/45 s	55°C/15 s	72°C/30 s	72°C/10 min	(26)
5	*tst*	95°C/5 min	95°C/2 min	54°C/2 min	72°C/2 min	72°C/10 min	(27)
6	*eta*	95°C/5 min	95°C/2 min	54°C/2 min	72°C/2 min	72°C/10 min	(27)
7	*arcA*	95°C/5 min	94°C/20 s	55°C/30 s	72°C/30 s	72°C/10 min	(28)

a *Temp, Temperature*.

b *min, Minutes*.

c *s, Seconds*.

#### PCR Identification of Staphylococcal Genes for Virulence Factors

Genes for virulence factors, namely: PVL cytotoxin genes *(lukS* and *lukF*), TSST-1 (encoded by *tst* gene), ETA (encoded by *eta*), and *arc*A (a surrogate marker for ACME I in the *arc* gene cluster) were tested using PCR for all isolates. These virulence factors and staphylococcal toxin genes were identified by singular PCRs with amplicons of: 433, 350, 93, and 724 bp, respectively (Table 1) (26–28).

#### PCR Assay and Visualization

All PCR reactions were optimized and carried by the Basic Gradient Thermocycler (BiometraTProfessional, Jena, Germany)using ready mix (Syntezza, Israel) according to the manufacturer's instructions, (Table 2) (29). Amplicons for all the characterized genes were analyzed electrophoretically in 2% agarose gels and visualized by UV light using a gel documentation system (Bio-Imaging Systems Mini-Lumitransilluminator, Germany).

### Statistical Analysis

All data were analyzed using the Statistical Package for Social Sciences (SPSS) version 20, using Chi-square test. A *p*-value of < 0.05 was considered to be statistically significant.

## Results

### Bacterial Isolates and Study Population

From November 2015 to July 2017, a total of 112 MRSA isolates were collected from major hospitals in the West Bank-Palestine. The majority of isolates was collected from Al-Makassed Islamic Charitable Hospital (*n* = 77, 68.8%). Place of residence for all included patients were documented. The most frequent regions of residence were patients from Gaza and Jerusalem (29.5% for each), followed by Hebron (13.4%), Nablus (10.7%), Ramallah (10.7%), Bethlehem (4.5%), Tubas (0.9%), and Tulkarem (0.9%). According to gender, sixty MRSA isolates (53.6%) were obtained from males and 41(36.6%) were obtained from females. Gender of 9.8% of the isolates was not reported. The mean age was 33 years with the oldest case being an 85 years old female and the youngest a 2 months years old female. The most common age group was adults (60.7%), followed by adolescents (14.3%), infants (8.9%), and children (7.1%). Of the adults, most were older than 30 years old, while age was not found for 8.9% of the samples. Samples were collected from different clinical sources. The highest number of isolates were obtained from wound infections (35.7%), followed by: blood culture (12.5%), nasal swabs (8.9%), urine culture (8.9%), pus culture (5.4%), tissue culture (4.5%), abscess (3.6%), ear swabs (2.7%), and sputum culture (2.7%). One MRSA isolate was collected and reported from a CSF culture (0.9%) which is extremely alarming and important in which MRSA strains are reaching the CSF leading to significant bacterial meningitis. Moreover, MRSA was obtained from other sources (4.5%), as the follows: axillary swab (0.9%), CVP culture (0.9%), skin swab culture (0.9%), synovial fluid culture (0.9%), and trap (0.9%). Source of eleven isolates (9.8%) was not identified.

### Detection of MRSA by Cefoxitin Disk Diffusion Test and *mec*A Gene

All isolates (100%) were verified as Methicillin Resistant *Staphylococcus aureus* using the cefoxitin disc resistance (<22 mm) and confirmed as MRSA using PCR targeting the *mec*A gene yielding to 310 bp clear band.

### Antimicrobial Susceptibility Patterns

**Figure 1:**
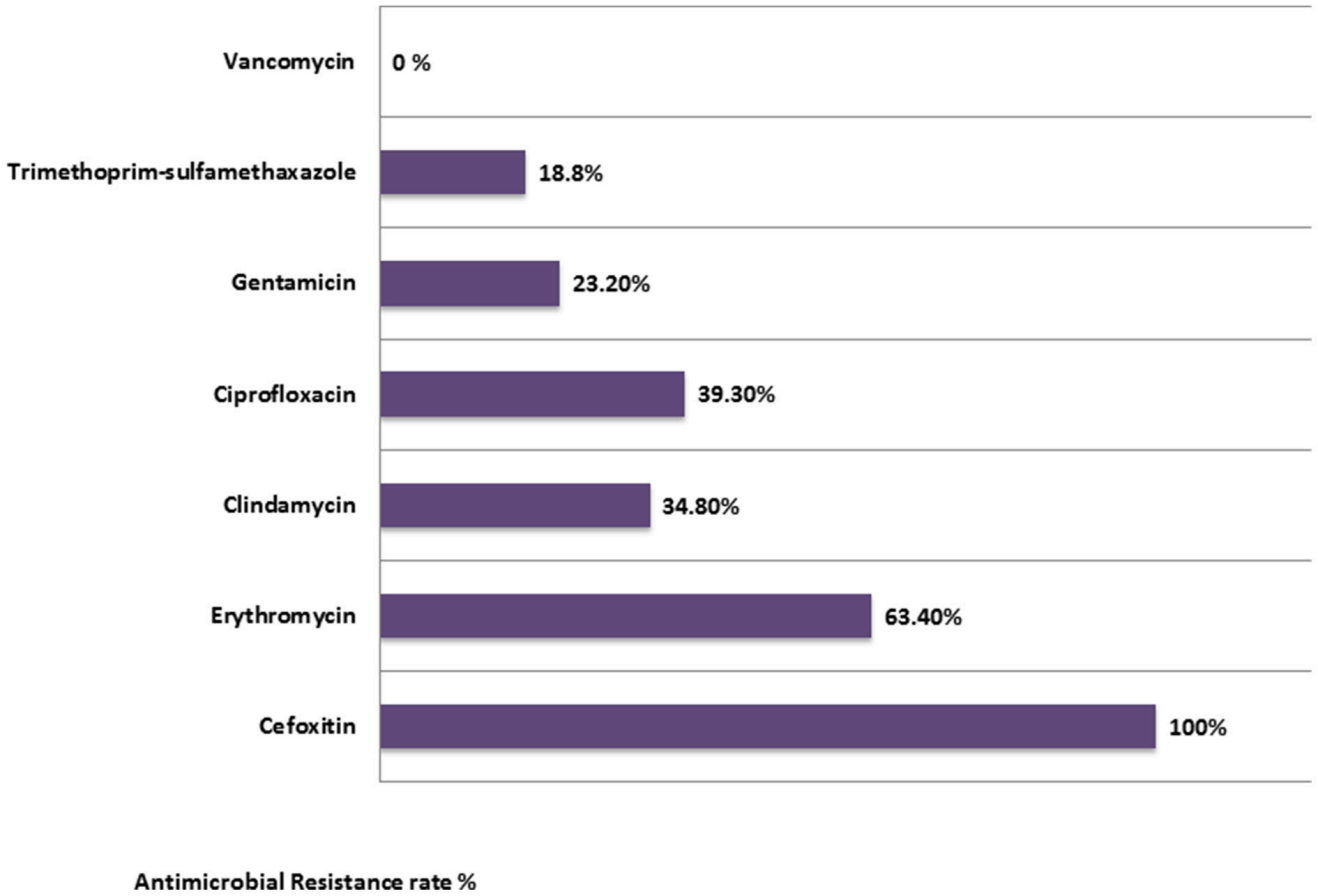
Antimicrobial resistance rates among all MRSA isolates in this study.

Resistance rates to non β-lactam antibiotics were as the following: 18.8% were resistant to trimethoprim-sulfamethoxazole, 23.2% were resistant to gentamicin, 34.8% to clindamycin, 39.3% to ciprofloxacin, and 63.4% to erythromycin. Interestingly, the susceptibility of MRSA isolates to gentamicin and Trimethoprim/sulfamethoxazole (SXT) was the highest (76.8, 81.2%), respectively. All isolates were susceptible to Vancomycin (100%). Of all the isolates, 32 were MDR (Figure 1).

The antimicrobial resistance rates among the MRSA *spa* types were studied. The resistance rates of the MRSA isolates against erythromycin, were significantly high in the *spa* type t386 (*p* < 0.005), followed by the *spa* types: t008 (8.9%), t044 (5.4%), t021 (5.4%), and t223 (4.5%). Similarly, the resistance rates of MRSA isolates against clindamycin were high in the *spa* types: t386 (7.1%) and t008 (4.5%), followed by t044 (3.6%), and t021 (2.7%). Moreover, the resistance rates of MRSA isolates against ciprofloxacin and gentamicin were found significantly high (*p* < 0.05) in the *spa* type t037 (7.1% for each). Also, for the *spa* types t008 and t044, they have shown a significant high resistance rate to ciprofloxacin (*p* < 0.05).

### Molecular Characterization and Typing of Isolates

#### SCCmec Typing

Among all MRSA isolates, ninety seven isolates belonged to SCC*mec* type IV (86.6%) and one belonged to SCC*mec* type V (0.9%). These isolates identified as CA-MRSA when carrying the SCC*mec* types IV or V. Ten isolates belonged to SCC*mec* type I (8.9%) and identified as HA-MRSA. Both SCC*mec* type II and SCC*mec* type III were not detected among isolates. Four MRSA isolates (3.6%) could not be SCC*mec* typed and designated as non-typeable (NT).

#### Spa Typing

**Figure 2:**
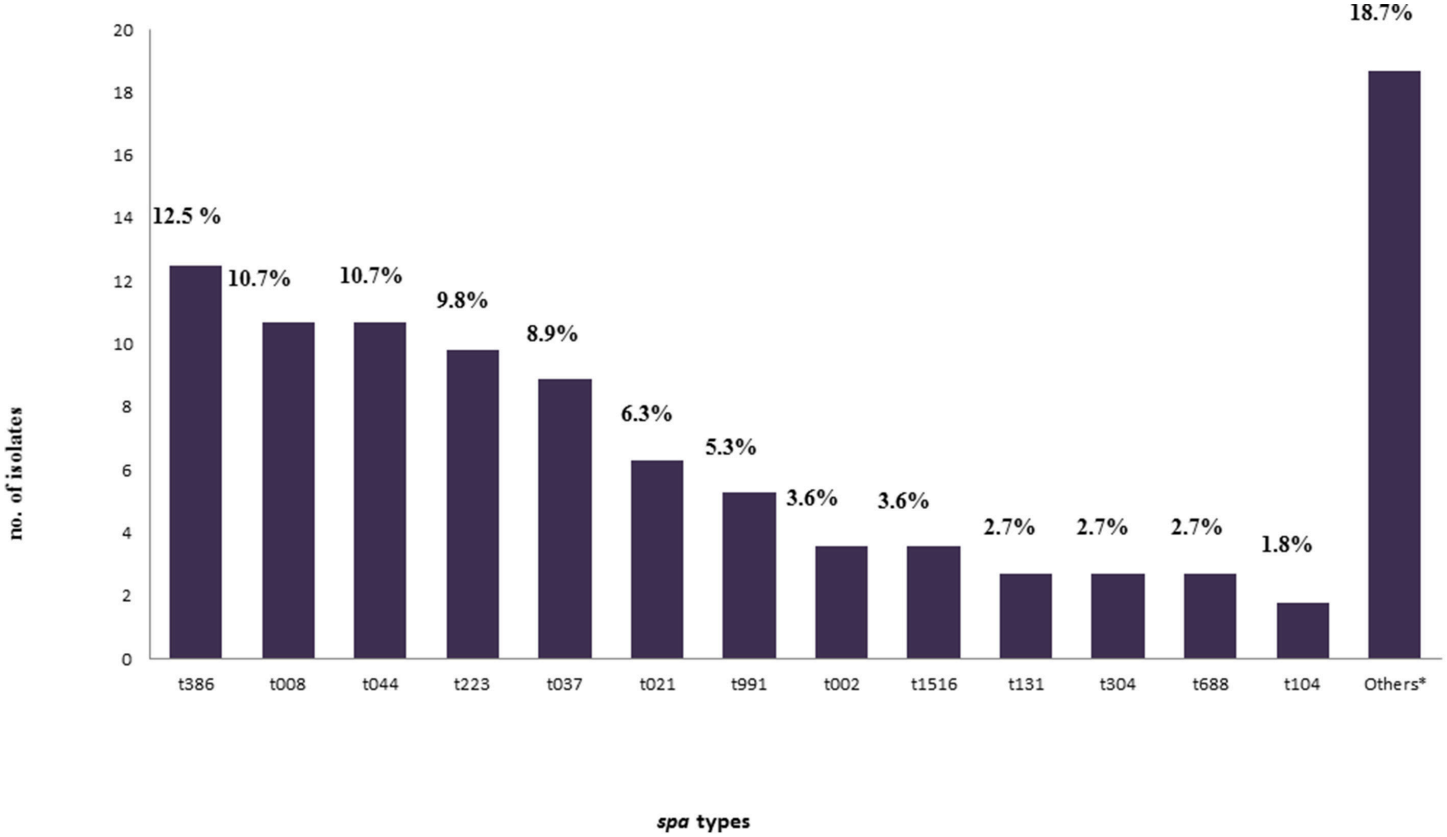
Frequency of all *spa*-types found in this study.*Other *spa*-types: t005, t011, t018, t084, t085, t1094, t121, t1247, t127, t314, t318, t359, t541, t605, t648, and t932 were found in a frequency of one for each isolate (0.9% for each) with four isolates (3.6%) non-typeable (err) isolates.

A total of twenty nine *spa* types were identified. The size of the amplified DNA for *spa* typing ranged between 200 and 450 bp. Five *spa* types: t386, t008, t044, t223, and t037 were predominant and represented (12.5%), (10.7%), (10.7%), (9.8%), (8.9%) of isolates, respectively (Figure 2). There was a wide range of clonal varieties, with twelve MLST clonal complexes (CCs) were identified. This was done with regard to the BURP analysis. The 12 identified MLST-CCs were: CC22, CC1, CC8, CC80, CC8/239, CC30, CC5, CC913, CC6, CC121, CC126, and CC15. The CC22 (15.2%), CC1 (13.4%), CC8 13.4%), and CC80 (13.4%) were predominant, followed by CC8/239 (8.9%), CC30 (8.0%), CC5 (8.0%), CC913 (5.4%), CC6 (2.7%), CC121 (0.9%), and CC126 (0.9%).Unfortunately, ten isolates (8.9%) were not found using the BURP analysis. However, the identified MLST-CCs were distributed among the *spa* types where a single clonal complex could contain more than one *spa* type as shown in Table 3.

**Table 3:** The MLST-CCs and the related *spa* types found in this study by the BURP.

**#**	**MLST-CC^a^s**	***spa* types**	**No. of isolates (%)**
1	CC22	t223	11(9.8%)
		t1516	4 (3.6%)
		t005	1 (0.9%)
		t541	1 (0.9%)
2	CC1	t386	14 (12.5%)
		t127	1 (0.9%)
3	CC8	t008	12 (10.7%)
		t121	1 (0.9%)
		t359	1 (0.9%)
		t648	1 (0.9%)
4	CC80	t044	12 (10.7%)
		t131	3 (2.7%)
5	CC8/239^b^	t037	10 (8.9%)
6	CC30	t021	7 (6.3%)
		t018	1 (0.9%)
		t318	1 (0.9%)
7	CC5	t002	4 (3.6%)
		t688	3 (2.7%)
		t104	2 (1.8%)
8	CC913	t991	6 (5.4%)
9	CC6	t304	3 (2.7%)
10	CC121	t314	1 (0.9%)
11	CC126	t605	1 (0.9%)
12	CC15	t084	1 (0.9%)
13	NF^c^	t011	1 (0.9%)
		t085	1 (0.9%)
		t1094	1 (0.9%)
		t1247	1 (0.9%)
		t932	1 (0.9%)
		(*)?	1 (0.9%)

(*)?, This spa-type was not identified on the spa database website; (http://spatyper.fortinbras.us/).

a *Clonal Complexes*.

b *The spa-t037 found to be CC8 or CC239 by the BURP, MLST is recommended for finite identification*.

c *Not Found by BURP*.

### Toxin Genes Profiling

#### Detection of PVL Toxin

Of all MRSA isolates, thirty three (29.5%) were positive for PVL toxin gene. All the PVL positive MRSA isolates belonged to SCC*mec* type IV. PVL was detected among eleven *spa* types: t044, t008, t223, t021, t002, t131, t104, t084, t121, t318, and t386. Two *spa* types (t044 and t008) among PVL positive isolates were predominant and represent (11/12 t044, CC80: PVL +; European clone) and (10/12 t008, CC8: PVL +; USA-300 clone) isolates, respectively. Out of 11 isolates of the *spa* type t223, only one isolate was positive for the PVL toxin gene (1/11 isolates t223, CC22: PVL +) and classified as a “Unique Gaza Strain.”

#### Detection of ACME-arcA Gene

The gene coding for the ACME-*arc*A was detected in 18.8 % (21/112) of isolates where 20 isolates harbored the SCC*mec* type IV, and one isolate harbored the SCC*mec* type I. It was detected among nine *spa* types: t008, t991 t037, t104, t1094, t1247, t1516, t223, and t386. Whereas, out of the 12 isolated identified as *spa* type t008, nine isolates were ACME-*arc*A positive (9/12 t008, CC8: ACME-*arc*A). These nine isolate were also PVL positive and classified as: USA-300 clone.

#### Detection of TSST-1

Similarly, 26.8% of the MRSA isolates (30/112) tested positive for the presence of the TSST- where 25 isolates belonged to the SCC*mec* type IV; while the other TSST-1 positive isolate belonged to SCC*mec* type I. This toxin was detected among eleven *spa* types: t223, t021, t1516, t002, 1005, t008, t018, t386, t541, t991, and t605. The two most common *spa* types among TSST-1positive isolates were: the *spa* type t223 and t021. Out of the 11 identified isolated as *spa* type t223, nine t223 MRSA isolates were significantly (*p* < 0.05) TSST-1 positive (9/11 t223, CC22: TSST-1 +; Gaza clone) which was commonly typed previously as “Gaza clone” in Gaza region. Notably, all the seven isolates with *spa* type t021 were TSST-1 positive (100%) and classified as South West Pacific clone.

Table 4 summarized the common MRSA clones with antibiotic resistance according to their SCC*mec* types, *spa* types and toxin genes profile.

**Table 4:** Common MRSA clones with antibiotic resistance according to their SCC*mec* types, *spa* types and toxin genes profile found in this study.

**MRSA Clone**	**n**	**%**	***spa* type/CC**	**SCCmec**	**Virulence factors**			^*^% Antibiotic resistance				
					**PVL**	**TSST-1**	***arcA***	**E**	**C**	**CN**	**CIP**	**SXT**
t386-IV	14	12.5	t386/CC1	IV	neg	neg	neg	100	57.1	21.4	21.4	0
The European clone	11	9.8	t044/CC80	IV	pos	neg	neg	50	33.3	16.7	41.7	16.7
The Gaza clone	10	8.9	t223/CC1	IV	neg	pos	neg	45.5	18.2	18.2	9.1	27.3
t037-IV	10	8.9	t037/ CC8/239	IV	neg	neg	neg	40	20	80	80	10
USA-300 clone	9	8	t008/CC8	IV	pos	neg	pos	83.3	41.7	0	75	8

* *:E, Erythromycin; C, Clindamycin; CN, Gentamicin; CIP, Ciprofloxacin; SXT, Trimethoprim sulfamethoxazole*.

#### Detection of ETA

The gene coding for the exfoliative toxin A (*eta*) was detected in 5.3% of all the MRSA isolates harboring the *spa* type t991/CC913. All the other *spa* types were negative for ETA. Among the 6 isolates with the *spa* type t991, five isolates were of SCC*mec* type IV, and one isolate was belonged to SCC*mec* type I. None of the *spa* t991; *eta* positive isolates were harboring the PVL toxin, and only one isolate was positive for ACME-*arc*A while 4/6 *spa* t991 isolates harbored the TSST-1.

## Discussion

Methicillin Resistant *Staphylococcus aureus* (MRSA) is a serious life threatening pathogen in hospitals and among healthy populations (29–32). Thus, the characterization of these strains is important for local epidemiology and surveillance studies.

Little is known about MRSA types and clones in Palestine. This study was conducted to characterize the molecular and the antimicrobial profile of MRSA isolates in regions of West Bank, from 2015 to 2017. Most isolates were obtained from Al- Makassed Islamic Charitable Society Hospital (68.8% of all isolates). Al-Makassed is one of the most important leading medical institutions in Palestine that provides medical services to all Palestinians in the West Bank, Gaza Strip and East Jerusalem.

In a recent study conducted at Al-Shifa hospital in Gaza Strip for the nasal colonization of MRSA, a carriage rate of MRSA among the health care workers who are in contact with the vulnerable patient in the hospitals were equal to 25.5% (33).This highlights that there is a high carriage rate of MRSA among the Palestinian populations. Moreover, it has been reported that carriage of MRSA is a major risk factor for transmission and subsequent infections that may develop to systemic or severe infections (34, 35).

Here, 32 MRSA isolates were MDR (28.6%). In comparison with other reports based on the same definition, a higher MDR resistance rate (60.0%) was also reported in Israel (36). However, our highest antibiotic susceptibility patterns were noticed among SXT (81.3%), followed by gentamicin (76.8%), and ciprofloxacin (65.2%). This may aid medical laboratory technologist to test the susceptibility of these antibiotics to be used as major and first line therapeutic options for the treatment of MRSA infections. No Vancomycin Resistance *Staphylococcus aureus* (VRSA) strains were reported, where the physicians can still prescribe this antibiotic based on empirical therapy when needed, especially for urgent infections. At the molecular level, the predominant MRSA strains in our region are community associated (SCC*mec* type IV, 86.6%). SCC*mec* type IV is the smallest structural type among the SCC*mec* types and believed to be the most mobile version that is associated with CA-MRSA infections (37). About one third of the CA-MRSA strains isolated in our study were harboring the PVL toxin gene (32/97 SCC*mec* type IV). This indicates that the CA-MRSA strains among Palestinian population are highly virulent with invasive. This can give us an indication that we may have a high carriage of MRSA among healthy population or the health care workers in our region. These CA-MRSA strains could be carried as a normal flora in skin, hands, and groin or in the nasal cavity of the health care workers or the patients themselves, but when these patients are hospitalized or have an opened wound or surgery, their immunity may fall down allowing this normal flora or colonization to cause secondary opportunistic infections. Therefore, hand hygiene and infectious control programs must be applied well in our hospitals. This interface may serve as a causative agent of cross contamination of hospital acquired and community acquired MRSA infections.

This agreed with a study done in Copenhagen where Bartels and his colleagues reported that there is a rapid shift to CA-MRSA, where SCC*mec* type IV was found in (86%) of isolates (38). Moreover, a significant presence of MRSA was seen. In this study, 62 hospitals were included showing an evidence of endemicity of MRSA in these regions (39). Also, it has been reported that there is an introduction of several MRSA strains with intercontinental exchange of new MRSA clones in the Middle East (40). In a recent study conducted in Gaza by Al Laham and his colleagues, similar results were obtained with a high frequency of the SCC*mec* type IV, which accounted for (79.3%) of MRSA isolates. Al Laham has found that the most dominant *spa* types were t223 and t044 where seven isolates were belonged to the *spa* type t223, in which one of these isolates was called as a “unique Gaza strain” harboring the PVL toxin gene, while all the other isolates with the *spa* type t223 did not carry the PVL toxin gene (19). This was in a high agreement with our findings with an isolate with a similar pattern as the unique Gaza strain with *spa* type t223, SCC*mec* type IV, PVL positive, and TSST-1 negative. Notably, the Gaza clone (t223) with TSST-1 positive, CC22, and SCC*mec* type IV was detected in the isolates collected from Al- Makassed Islamic Charitable Society Hospital only. Six isolates were from Gaza patient and five isolates were from patients living in Jerusalem and its suburbs. Interestingly, this could be explained due the transmission of the Gaza clone to other Palestinian populations as Gaza patients were referred to Al- Makassed Islamic Charitable Society Hospital in Jerusalem. Moreover, the *spa* type t223 was also reported in other countries as Kuwait, Egypt and Saudi Arabia. This transmission could be due to the national travels between these countries (41–45).

The *spa* type t008 was predominant in 12 MRSA isolates. Nine of them were related to the USA-300 clone, harboring both the PVL and the ACME-*arc*A genes (8% of all isolates). This clone is a common cause of SSTIs and was considered as the most widespread CA-MRSA clone in the United States that emerged in the late 1990s. This clone is a significant and dramatic epidemic clone due to its carriage of virulence and resistance determinants that may enhance severity and pathogenicity of the isolated strain (17). The carriage of PVL toxin gene among the CA-MRSA strains, especially the USA-300 clone, is crucial and make these strains hyper-virulent which may cause occasionally fatal infections (46). To the best of our knowledge, our study is the first study that has characterized the presence of the USA-300 clone in the Palestinian regions. Also, due to the shift of MRSA infections to the community and the high carriage among populations, no individual can be considered not at risk for CA-MRSA infection. This worthy point needs further attention because meaningful results and control programs can be achieved. Regarding the appropriate treatment approaches, culturing is recommended for the MRSA infections because it is the only ideal predictor of the appropriate antibiotics therapy. Educational and public awareness, medical education, professional development, and training are recommended for the public society, parents, pharmacists, medical laboratories and physicians for reliable, appropriate and proper antibiotic therapy decisions. This can help to minimize the development for more complicated resistant MRSA strains in hospitals and may save many immunocompromized hospitalized patients. All together, these points suggest the need for efficient future surveillance studies and infection control strategies. Moreover, these strategies can improve the economic impact because MRSA is considered as a serious economic burden on the healthcare resource and associated with increased hospitalization costs due to the prolonged course on more complex antibiotics and extended hospital days.

The results of this study showed that all the PVL-positive MRSA isolates (29.5% of all isolate) recovered from these hospitals as part of the present investigation were CA which is confirming as an earlier preliminary observation that CA-MRSA is an emerging problem in Palestine. Some reports have suggested that certain strains of CA-MRSA may be more virulent than HA-MRSA (47, 48). The expression of PVL, cytolytic toxin that targets mononuclear and polymorphonuclear cells and causes cell death by necrosisor apoptosis, has been strongly linked with CA-MRSA (49).

Similarly, TSST-1 and ACME-*arc*A genes were detected in 23.2 and 18.8% of all isolates, respectively. These were relatively high in comparison with a similar study conducted in China where they found the PVL and TSST-1 genes in 11.3 and 18.8%, respectively (50). For the ACME-*arc*A carriage, this was in agreement with a study conducted in Armenia which found the ACME-*arc*A in 15% of all MRSA isolates, but contraindicated with the carriage of the PVL toxin gene where they did not detected the PVL toxin gene in any of their MRSA isolates (51). This indicates that there is a high carriage of PVL, ACME-*arc*A, TSST-1, and ETA genes among MRSA strains in Palestine.

## Conclusion

The phenotypic and genotypic diversity with harboring several virulence factors and toxin genes by MRSA isolates in Palestine is crucial. This study indicates that we have unique MRSA strains which may be associated with more complicated infections and associated with more social and economic burdens. Thus, there is an urgent need to develop better measurements among clinical microbiology laboratories for proper detection, identification, and reporting of MRSA isolates with deep knowledge among physicians toward antibiotic stewardship and prescription practices for this multi-drug resistant organism. These together may control and limit the development and spread of new clones and more complicated MRSA infections.

## Ethics Statement

The study was approved by the Research Ethical Committee at Al-Quds University. Written informed consents were sent for the participating hospitals and clinics. Written informed consent was obtained from the parents of the participants that were under the age of 16 years old.

## Author Contributions

KA designed the study and wrote the final manuscript. EH and KA performed the experiments, analyzed the obtain results. RA and IA participated in the sample collection. ZA contributed to the manuscript revision and overall support of this study.

### Conflict of Interest Statement

The authors declare that the research was conducted in the absence of any commercial or financial relationships that could be construed as a potential conflict of interest.
